# Implementation of social needs screening in primary care: a qualitative study using the health equity implementation framework

**DOI:** 10.1186/s12913-021-06991-3

**Published:** 2021-09-17

**Authors:** Connor Drake, Heather Batchelder, Tyler Lian, Meagan Cannady, Morris Weinberger, Howard Eisenson, Emily Esmaili, Allison Lewinski, Leah L. Zullig, Amber Haley, David Edelman, Christopher M. Shea

**Affiliations:** 1 Department of Population Health Sciences, Duke University School of Medicine, Durham, NC USA; 2 Center for Personalized Health Care, Duke University School of Medicine, Durham, NC USA; 3 Department of Health Policy and Management, Gillings School of Global Public Health, University of North Carolina at Chapel Hill, Chapel Hill, NC USA; 4 Lincoln Community Health Center, Durham, NC USA; 5 Global Health Institute, Duke University, Durham, NC USA; 6 Duke University School of Nursing, Durham, NC USA; 7 Center of Innovation to Accelerate Discovery and Practice Transformation (ADAPT), Durham Veterans Affairs Medical Center, Durham, USA; 8 Department of Medicine, Duke University School of Medicine, Durham, NC USA

**Keywords:** Social determinants of health, Social needs, Health equity implementation framework, Primary care, PRAPARE

## Abstract

**Background:**

Screening in primary care for unmet individual social needs (e.g., housing instability, food insecurity, unemployment, social isolation) is critical to addressing their deleterious effects on patients’ health outcomes. To our knowledge, this is the first study to apply an implementation science framework to identify implementation factors and best practices for social needs screening and response.

**Methods:**

Guided by the Health Equity Implementation Framework (HEIF), we collected qualitative data from clinicians and patients to evaluate barriers and facilitators to implementing the Protocol for Responding to and Assessing Patients’ Assets, Risks, and Experiences (PRAPARE), a standardized social needs screening and response protocol, in a federally qualified health center. Eligible patients who received the PRAPARE as a standard of care were invited to participate in semi-structured interviews. We also obtained front-line clinician perspectives in a semi-structured focus group. HEIF domains informed a directed content analysis.

**Results:**

Patients and clinicians (i.e., case managers) reported implementation barriers and facilitators across multiple domains (e.g., clinical encounters, patient and provider factors, inner context, outer context, and societal influence). Implementation barriers included structural and policy level determinants related to resource availability, discrimination, and administrative burden. Facilitators included evidence-based clinical techniques for shared decision making (e.g., motivational interviewing), team-based staffing models, and beliefs related to alignment of the PRAPARE with patient-centered care. We found high levels of patient acceptability and opportunities for adaptation to increase equitable adoption and reach.

**Conclusion:**

Our results provide practical insight into the implementation of the PRAPARE or similar social needs screening and response protocols in primary care at the individual encounter, organizational, community, and societal levels. Future research should focus on developing discrete implementation strategies to promote social needs screening and response, and associated multisector care coordination to improve health outcomes and equity for vulnerable and marginalized patient populations.

**Supplementary Information:**

The online version contains supplementary material available at 10.1186/s12913-021-06991-3.

## Introduction

Social determinants of health (SDOH) are defined by the World Health Organization as the conditions in which people are born, grow, work, live, and age and encompass the wider set of forces and systems shaping conditions of daily life. This includes the effect of economic policies and systems [1] and manifest as downstream patient-level social needs (e.g., employment, safety, food security, social support, transportation). Due to how these social needs are major drivers of health outcomes [2, 3], disparities [4, 5], and health care utilization [6–9], health care systems are increasingly identifying and implementing strategies to address these modifiable unmet needs. Through embracing financial incentives to deliver value-based care [10, 11] and improving management of population health [12], health care systems have increased their uptake of screening and response (S/R) approaches [13, 14] to further enhance how social care is integrated into routine clinical encounters [15].

Social needs S/R protocols are utilized globally to improve population health [16, 17]. Patients are administered a brief questionnaire to screen for unmet social needs and refer them to community-based organizations, social service agencies, or internal programs for needed assistance, resources, or services. The Centers for Medicare and Medicaid Services and state Medicaid agencies have incorporated social needs S/R as an element of care innovation [11, 18, 19]. In a 2018 survey analysis, 24% of hospitals and 16% of US physician practices reported screening for social needs [20]. The Protocol for Responding to and Assessing Patients’ Assets, Risks, and Experiences (PRAPARE) is the most common social needs S/R protocol used by United States hospitals, health systems, and health plans [21]. The PRAPARE is part of a national effort to collect data to understand the upstream SDOH drivers of poor health outcomes and higher health-related costs. Responding to social and nonmedical needs is consistent with chronic care frameworks for delivering high-quality, patient-centered care [22–26].

Current literature describing efforts to screen and respond to social needs has focused on electronic health record (EHR) integration [27–29], provider perspectives [30–33], and patient acceptability, and has sometimes used implementation case studies [34–36]. This existing literature suggests that patients consider social needs S/R an acceptable and important element of patient-centered care, but they recognize the limits of the health care sector’s capacity to resolve unmet social needs. A 2018 systematic review of eight studies found that leadership, management, and local infrastructure were important implementation factors but concluded that much remains unknown about factors contributing to effective implementation [37]. Despite implementation science theories and frameworks that emphasize the importance of patient, client, or recipient perspectives as determinants of successful implementation, the literature lacks the application of an implementation science framework to describe stakeholder perspectives on barriers and facilitators associated with integrating social needs S/R protocols into outpatient clinical encounters.

An understanding of barriers and facilitators would contribute to the growing body of literature supporting the adoption of the PRAPARE or similar approaches in diverse clinical settings [11, 38–41] and inform care models designed to promote health equity by mitigating disparities for vulnerable or marginalized groups whose unmet social needs are rooted in economic exclusion, historic marginalization, and racism. This study identifies the barriers and facilitators to implementing a S/R protocol for unmet social needs reported by patients and clinicians in federally qualified community health center (FQHC) clinics.

## Methods

### Study setting

The study site was a FQHC in a medium-sized southeastern U.S. city. It is accredited as a primary care medical home by The Joint Commission and served ~ 36,000 unique patients across nine outpatient and community-based clinical sites in 2019. Services include primary care medical clinics (Pediatrics, Adult Medicine, and Family Medicine); a Behavioral Health Clinic integrated with primary care; a dental clinic; a Women, Infants, and Children (WIC) clinic; a pharmacy with medications at discounted prices; a laboratory; and a radiology unit. The FQHC provides a care coordination team, case managers (behavioral health team members), Spanish interpreters (telephone interpretation for other languages), and free van transportation to and from appointments. The FQHC employs over 200 people and is governed by a community Board of Directors with majority representation by patients. Over 92% of patients are underrepresented racial or ethnic minorities, 56.8% are uninsured, and 97.1% are below 200% of the federal poverty limit.

### Social needs screening and response

Social needs S/R was based on the FQHC’s standardized PRAPARE practice patterns: (i) patient identification, (ii) screening, and (iii) response through referral to in-house resources, community-based organizations (CBOs), and/or social service agencies. Patient identification for social needs S/R in the FQHC occurs via a primary care provider referral to the behavioral health team (via warm hand-off or separate scheduled visit) or as part of the intake process for a behavioral health visit. Trained social workers are the clinicians that identify patients’ social needs using the PRAPARE assessment tool [42] and makes referrals to a broad range of resources through the FQHC (e.g., medication assistance vouchers and transportation assistance), local community (e.g., CBOs that provide shelter or food), and entitlements or public programs (e.g., Supplemental Nutrition Assistance Program or Medicaid). Patients may receive follow-up and case management to ensure that they receive resources for which they have been referred. Additional details of the clinical delivery of the PRAPARE at the participating clinical site have been published [43].

### Health equity implementation framework

**Fig. 1:**
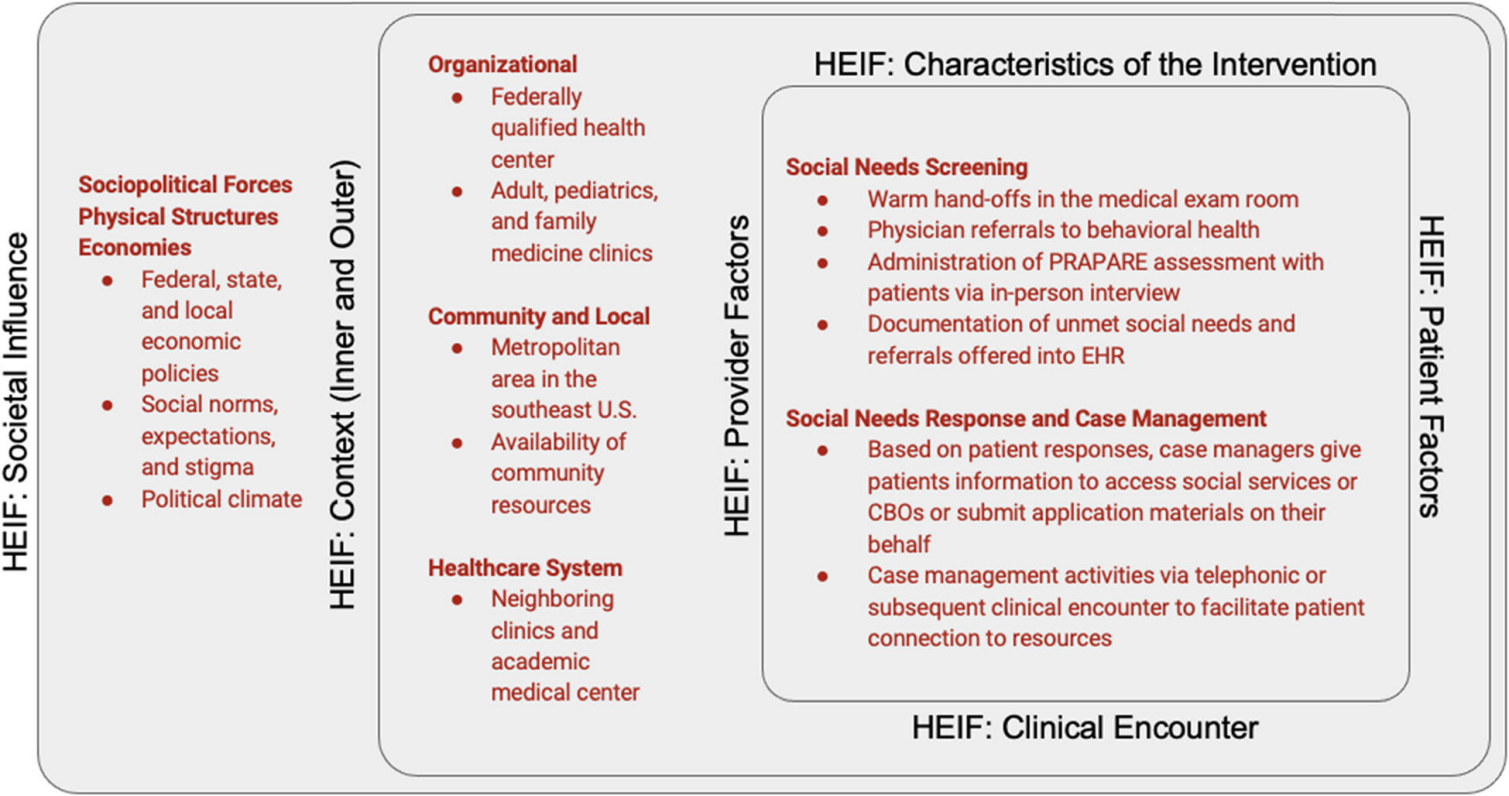
HEIF constructs and social needs screening and response activities

Qualitative research methods have an important role in implementation research. Designed primarily to understand ‘how’ an innovation works in practice, qualitative methods investigate factors that surround and interact with implementation processes [44]. To understand how these contextual factors support or hinder implementation, we used the health equity implementation framework (HEIF) to identify and evaluate existing barriers and facilitators to implementation of the PRAPARE tool at the FQHC [45]. HEIF integrates two frameworks: (i) the Integrated-Promoting Action on Research Implementation in Health Services (i-PARIHS), which accounts for multiple levels of implementation determinants (context or systems level, recipients, and characteristic of the innovation or intervention) and posits that the most effective implementation strategies must be multifaceted to account for these distinct levels; and (ii) the Health Care Disparities Framework to identify drivers of health disparities at the patient, provider, clinic, and health system level. We chose the HEIF to qualitatively evaluate social needs S/R implementation because it accounts for both factors at multiple levels and implementation considerations that are unique to populations that are vulnerable due to social context and historical marginalization (Fig. 1).

The HEIF consists of five overarching domains: (i) *Characteristics of the Innovation*, (ii) *Clinical Encounter*, (iii) *Patient & Provider Factors*, (iv) *Inner & Outer Context*, and (v) *Societal Influence*. *Characteristics of the Innovation* includes the extent to which the approach is simple or intuitive and considered beneficial or useful. Implementation considerations related to the *Clinical Encounter* domain (i.e., clinical workflows leading to interactions between patient and provider or between recipients) provide unique characteristics and preferences affecting engagement with vulnerable populations. *Patient & Provider Factors* are specific to a patient or member of the health care team (e.g., beliefs, acceptance, privacy concerns, situation, training and knowledge, communication or engagement preferences, and attitudes towards relevant stakeholders or institutions). The *Inner & Outer Context* domain comprises forces more broadly related to health care (*Outer Context*), and the formal policies, previous experiences, or descriptions of drivers specific to the clinic (*Inner Context*) that have influenced implementation of clinical screening activities. Finally, *Societal Influence* (structural social and economic drivers) can significantly affect health care disparities and implementation drivers.

### Data collection

We invited patients to provide their perspectives on the PRAPARE through semi-structured interviews. Between November 2019 and February 2020, 10 patients participated in interviews with a trained member of the study team who had qualitative research experience (CD & HB). We used a purposeful criterion-sampling strategy to identify information-rich cases and ensure diversity by age, gender, and race [46]. Only English-speaking adult patients who received the PRAPARE as part of standard of care in the Adult Medicine, Family Medicine, and Pediatrics clinics were eligible to participate. Patients were invited to participate in the semi-structured interviews via in-person referral during the clinical encounter in which they were screened for unmet social needs. In November 2020, all five case managers delivering the PRAPARE within the three clinics were invited to participate in a virtual semi-structured focus group. Clinicians were invited to participate in the focus group via e-mail. All patients and clinicians invited to participate in the study accepted. We used the concept of “information power” to determine the sample size. As Malerud et al. suggest, the more information power the sample holds, the smaller the sample size needs to be. Information power is affected by five factors: study aim, sample specificity (i.e., specificity of experiences), use of established theory (i.e., HEIF), quality of dialogue in the interviews/focus group, and analysis strategy [47]. When appraising these factors before and during data collection, we found that the planned recruitment targets were appropriate for the research question of interest. The study team tasked with carrying out the qualitative data collection, design, and analyses consisted of trained researchers as part of a long-term academic-community partnership to evaluate and maintain social needs S/R activities. This partnership developed over a three-year period and included a multi-method evaluation. Consistent with the principles of community engagement, the priorities of the evaluation resulted from inclusive participation of FQHC leadership and stakeholders to ensure findings were relevant to practice and complementary to the organization’s values and goals [48]. The study team met regularly with clinic leadership and frontline staff stakeholders to determine research priorities, with an emphasis on informing equitable, patient-centered care practice patterns. As a result, the study team members were familiar with the five clinical case managers but had no prior relationship with the interviewed patients.

We designed a semi-structured interview guide to identify and evaluate barriers and facilitators across the HEIF domains (See Additional file 1). After patients had provided informed consent, a trained study team member (CD & HB) conducted interviews at the clinical site ranging from 40 to 75 min. Study team members (CD & TL) also led a focus group for clinicians to identify areas of consensus and explore issues in which there were contrasting opinions (See Additional file 2). Efforts were made to ensure confidentiality of responses in an environment free from the presence of a manager or clinical supervisor who could influence or censor responses. Clinician participants were purposively selected as information-rich cases based on their first-hand experience with the PRAPARE and relevant training. The clinician focus group lasted approximately 80 min and was conducted virtually due to the COVID-19 pandemic. The focus group and interviews were recorded on an encrypted recording device or secure audio-video software and then transcribed. Transcriptions were deidentified and stored in a secure study drive to only be accessed for analysis by members of the study team. Study participants were provided with an incentive to participate in the interviews and focus group. This study protocol was reviewed and approved by the Duke Health Institutional Review Board (Pro00093941). Per IRB instruction, subjects’ verbal or written consent were obtained prior to study participation and included permission to publish and disseminate study findings, including de-identified quotes. Patients who completed semi-structured interviews were provided a monetary incentive for their time via a pre-paid credit/debit card. To reduce the potential for bias and influencing responses, interviewers were not employed by the FQHC where patients received care. Moreover, patients were informed that their responses would be kept confidential and that only aggregate information would be shared to help inform implementation and disseminate results. Clinicians who participated in the focus groups were provided a meal during the focus group but no monetary incentive; they were also told that their responses would be kept confidential.

### Analytic plan

We used the standards for reporting qualitative research (SRQR) checklist to organize and report qualitative data [49]. A directed content analysis approach was used to analyze interview and focus group transcripts [50]. An initial codebook was created using a priori codes developed from the HEIF. To create the initial codebook, two trained coders (HB & MC) independently coded the patient interview transcripts linked to the relevant theoretical HEIF domains. This process was repeated with the focus group transcript by two trained coders (HB & TL) to further refine and update the codebook. Regular meetings were held among coders to confirm the definition and meaning of new categories and codes within the HEIF domains. Specifically, categories and codes either offered a distinct view of the phenomenon or served to further contextualize, expand, and enrich understanding of barriers and facilitators to implementation. Once the final codebook had been created, all transcripts were re-analyzed with the revised codebook. Coders met regularly to ensure consistent application of codes and reconcile differences through discussion or by revisiting transcripts for additional context. A separate coder (CD) was included in discussions to resolve coding differences in order to finalize the application of codes across transcripts. All coding and analysis were conducted using NVivo version 12.6.0.

## Results

Semi-structured interviews were conducted with 10 patients (5 females and 5 males) ranging in age from 26 to 64 years. Seven patients were uninsured, and 8 indicated that English was their preferred language. The majority lived in a household consisting of 1–4 people and felt safe in their home. They reported a variety of unmet social needs during screening, including food insecurity, lack of health care access, unemployment, stress or emotional needs, transportation challenges, and other material needs (e.g., financial assistance for utilities). Their reports were broadly representative of common social needs in a larger patient population that had been screened for social needs based on available EHR data (*n* = 2192) (Table 1). All five clinicians participating in the focus group had social work training and had been in clinical practice for 3–14 years (Table 2). To facilitate interpretation, we organized the reported barriers and facilitators around HEIF domains within screening and referral clinical activities (Table 3). Descriptions of HEIF domains with illustrative quotes of the associated implementation barriers and facilitators are provided in Table 4.

**Table 1:** Semi-structured interview patient participant demographics

	Interview Participants (***n*** = 10)	Screened Patient Population (***n*** = 2192)
**Age at Encounter**		
18–44 years	30.0 (3)	36.1 (792)
45–60 years	40.0 (4)	40.5 (887)
61+ years	30.0 (3)	23.4 (513)
**Sex**		
Female	50.0 (5)	61.8 (1354)
Male	50.0 (5)	38.2 (838)
**Race and Ethnicity**		
Black, non-Hispanic/Latino	80.0 (8)	49.2 (1078)
Hispanic/Latino	20.0 (2)	35.1 (769)
**Number in Household**		
1–4	90.0 (9)	73.6 (1614)
5+	10.0 (1)	16.7 (365)
**Education Level**		
Less than high school degree	10.0 (1)	32.2 (705)
High school diploma or GED	10.0 (1)	31.5 (691)
More than high school degree	80.0 (8)	29.2 (640)
**Current Work Situation**		
Unemployed	10.0 (1)	24.6 (540)
Part-time or temp work	20.0 (2)	18.1 (397)
Full-time work	20.0 (2)	23.0 (504)
Unemployed, not seeking work	40.0 (4)	26.8 (588)
Non-response	10.0 (1)	7.4 (163)
**Primary Insurance**		
None/uninsured	70.0 (7)	54.2 (1188)
Medicaid	20.0 (2)	12.9 (283)
Medicare	10.0 (1)	13.3 (291)
**Preferred Language**		
English	80.0 (8)	58.8 (1289)
Spanish	20.0 (2)	25.2 (553)
**Identified Social Needs**		
No housing	20.0 (2)	18.1 (396)
Worried about losing housing	10.0 (1)	13.3 (292)
Transportation barriers	10.0 (1)	16.1 (353)
Social isolation^a^	10.0 (1)	13.9 (305)
High stress^b^	30.0 (3)	22.5 (494)
Unsafe in neighborhood	10.0 (1)	7.2 (157)
**Received Referrals Categories**		
Food	10.0 (1)	14.1 (308)
Transportation	20.0 (2)	6.2 (136)
Housing	30.0 (3)	3.6 (80)
Financial	50.0 (5)	9.3 (204)
Access to medical care	40.0 (4)	17.4 (382)
Social and emotional health	20.0 (2)	7.8 (170)

^a^Refers to patients that indicated less than 1–2 weekly social support interactions per PRAPARE assessment

^b^Refers to patients that indicated ‘Quite a bit’ or ‘Very much’ when asked about stress levels per PRAPARE assessment

**Table 2:** Focus group clinician participant demographics

Variable	Number of Participants (***n*** = 5)
**Sex**	
Female	5
Male	0
**Race and Ethnicity**	
Black, non-Hispanic	1
White, non-Hispanic	1
Hispanic	2
Declined to report	1
**Education**	
MSW	5
**Licensure**	
LCAS-A	2
LMHC	1
LCSW	1
LCSW-A	1
**Languages**	
English	5
Spanish	2
**Years of Practice**	
1–10 years	4
11+ years	1
**Years at Partnering FQHC**	
1–3 years	4
4+ years	1

**Table 3:** HEIF domains and summary of key points

HEIF Domain	Description	Screening	Response
*Characteristics of the Innovation*	Characteristics related to the evidence-based approach: (in this case) both the PRAPARE screening and response protocols and any ongoing case management. Includes being simple or intuitive (*Ease of Use*) or beneficial or useful (*Effectiveness*), and how the integration of patient preferences and amount of time (Time) affected usability (Patient Preferences).	Patients appreciated the timing and convenience of the social needs screening, often inserted between encounters with clinicians; however, additional administrative responsibilities were time-consuming for clinicians.	Accurate and up-to-date information about community resources enabled a timely and tailored referral response to identified social needs.
*Clinical Encounter*	The clinical encounter or workflows that led to interaction between patient and provider or between recipients. These delivery mechanisms are of particular importance to vulnerable populations due to their unique characteristics or preferences for engagement.	Empathetic communication and rapport between patients and clinicians helped patients to feel comfortable with sharing their social needs.	Establishing rapport and trust during the clinical encounter facilitated patient acceptance of referrals in response to identified social needs.
*Patient Factors*	Patient-specific factors that affect implementation, including the individual’s beliefs, acceptability, privacy concerns, situation, preferences for communication or engagement, and attitudes towards relevant stakeholders or institutions.	Patients viewed social needs screening as not only an acceptable but an essential and appropriate component of high-quality, patient-centered care.	The co-occurrence of various social and medical conditions complicated the process of accessing services for patients and of making referrals for clinicians.
*Provider Factors*	Factors specific to the provider or care team including methods or techniques used to screen and respond for social needs (*Methods of Communication*); implementation considerations related to treatment, care planning, and addressing medical needs (*Treatment*); clinical techniques utilized during PRAPARE administration (*Clinical Training)*; and provider beliefs regarding the role, purpose, and characteristics of the PRAPARE, or delivery style (*Provider Beliefs*).	Clinicians expressed that the overall goals of social needs screening aligned with their professional goals and that patient engagement techniques from their clinical training were integral to its effective administration.	Clinicians’ knowledge of and familiarity with available community resources facilitated social needs response.
*Context*	Inner context factors include formal policies, previous experiences, or descriptions of political, social, or economic implementation drivers associated with the clinic, community based organizations, or locally administered social services. Outer context factors include engagement with health care systems at large or factors associated with access, quality, or institutional policies.	Sustainable screening processes required clinician perspectives to be incorporated into organizational decisions regarding screening workflows, compatibility with current workloads, and design of the screening assessment.	Staffing and time constraints strained clinicians’ efforts to manage a wide range of responsibilities while providing high-quality patient care. Fragmentation between health care systems also hindered care coordination.
*Societal Influence*	A description of factors that affect implementation but are subject to larger, structural forces including stigma, discrimination, societal expectations, economic policy, and/or political climate.	The appropriateness and effectiveness of screening depended on the availability of community resources to address identified social needs.	Structural inequities and scarcities in resource distribution impeded social needs response in specific domains such as housing. Administrative burden, means testing, and discrimination often prevented patients from accessing services.

**Table 4:** Respondents’ illustrative quotes describing implementation barriers or facilitators organized by the HEIF

HEIF Domain	Clinician-Reported Implementation Barriers and Facilitators	Patient-Reported Implementation Barriers and Facilitators
Characteristics of the Innovation	“I think part of that reason is- for me, it all depends on when, at which point, of the meeting I do the PRAPARE. If I do it towards the beginning and there’s really no rapport that has been built between me and the patient, they’re not going to be as open or as talkative. They’re not going to be as detailed with their answers. They may just give me “yes” or “no.” Or one word answers. But, because they don’t really know me, they don’t feel as comfortable. But, if I talk with them a little bit more or a little bit longer or if I’ve had previous visits with them and we have that rapport built already or even just a little bit of it, then, yeah, they’re going to open up with me more, and therefore I’m able to find out more details and needs that they have.” (African American female MSW, LCAS-A Case Manager with 4.5 years of practice experience; *Facilitator*) “For me, I guess I wanna know- I know how it’s [PRAPARE] helping patients identify barriers, life stressors, concerns, but what other way is it helping, really? Besides showing you, “Hey, this issue is here. We may be able to refer you out to a resource,” but it just feels like extra work. There’s no definite end for it. Like, how is it helping the patient, besides identifying that there is a problem?” (African American female MSW Case Manager with 14 years of practice experience; *Barrier*)	“I was just so grateful. I didn’t know I could get that kind of service the same day.” (62 year old African American female patient; *Facilitator*) “Another thing that <care manager name> told me was if I took the bus, that they could help with bus pass. <care manager name> was saying things like that. You know, she just threw out everything that could to help me.” (62 year old African American female patient; *Facilitator*) “I was like, very, very happy about that because sometimes—you know, I’ve been an independent person all my life. I never asked for help. I don’t like to ask when I can because somebody else is worse than me. And for her to go into what I was going through, for her to bring it out of me, to just say it—I was very grateful.” (62 year old African American female patient; *Facilitator*) “It was good because the food they gave me, it was something I could use. Like some pantries give you things and you really can’t use them, but they gave me things that I wouldn’t buy for myself like squash, zucchini. Sometimes I can’t really afford it, but they gave it to me.” (52 year old African American male patient; *Facilitator*)
Clinical Encounter	“So, I’ve come across a lot where, you know, a person may go through almost the whole entire PRAPARE with me, and they’re not identifying that they have any needs. But then, just kind of trying to talk to them and not administer the paperwork and just talking to them from a human being to a human being: that’s when Pandora’s Box gets opened up. And then we went from “no real needs” to “I need food. I need clothing. I’m trying to find a job. I don’t have internet.” (White/Caucasian female MSW, LCSW, LCAS-A Case Manager with 4 years of practice experience; *Facilitator*)	“…she took her time. I would say it was closer to eight. Maybe around eight to 10 min, something like that. She stood there. She took her time.” (52 year old African American male patient; *Facilitator*) “I was really impressed because she says, ‘Well, I left a message.’ She says, ‘If they don’t get back to you, call me and let me know.’ So, it just really impressed me. A lot of people say, ‘Oh, I’ll make a phone call. Somebody will get back to you.’ They never get—you know.” (52 year old African American male patient; *Facilitator*) *“…*I was surprised that she [clinician case manager] would even supply that kind of help, or just even tell me that I could get a little bit of help. She kept asking me, ‘After you got sick and went back to work, do you have food? Do you have money?’ She kept asking me those kind of questions now and I’m like, how did she even think about those kinds of things, because usually you go to your doctor and they would just treat you and you leave, but she was just awesome.” (62 year old African American female patient; *Facilitator*) “That’s what really just, you know, really just got to me. You know, I was telling you know the other clients, the other people that was sitting there, you know, about how well they helped, how quick—I mean they just—it really shocked me how quickly— They jumped in to help you.” (64 year old African American female patient; *Facilitator*)
Patient Factors	“I don’t know if it’s cultural-wise or not, but I think, with the Latinos, they don’t really often see another person like them screening and asking them, “Hey, do you need help?” So, when they do find someone, they’re going to open up and tell you, “Yeah, I need this and this and this.” Versus, maybe an American, who seems used to having multiple resources and people who speak their language – they can easily access it – versus a non-English speaker.” (Hispanic female MSW, LMHC Case Manager with 4 years of practice experience; *Facilitator)*	“Because a lot of people don’t realize how much help is out there. You know, unless you ask, you never know. And I’ve never dreamed of asking anybody at (FQHC name) that until she approached me that day. So, it really helped me, you know?” (58 year old African American male patient; *Barrier*) “For one, it shows that it’s not just about seeing a patient, getting them in and out, that they actually care about the patients. I’ve been going to (FQHC name) off and on for many years and I know that the majority of the patients, including myself, are low-income. So, knowing that there are other resources out that can help with different things, that’s real helpful.” (48 year old African American female patient; *Facilitator*) “No, I didn’t have any concerns about my privacy, because I felt like they was there to help, and you know, the only way for them to help me is to give them the information that they need to help me. If I beat around the bush, then it’ll take longer, because I’m not really giving them the straight information. So no, I really wasn’t concerned about, you know, my privacy.” (64 year old African American female patient; *Facilitator*)
Provider Factors	“I felt like, again, being clinically trained to be able to kind of recognize body language, if we came across a tough one like intimate partner violence one or- I could kind of see a change in their body language when I asked a question. Then that triggered me to kind of normalize what was going on and I found that that was a little bit more helpful for patients.” (White/Caucasian female MSW, LCSW, LCAS-A Case Manager with 4 years of practice experience; *Facilitator*) “…one of our main purposes in general, as far as the type of work we do, is to be able to see how the different parts of a person’s life impacts them because a lot of times one area influences the other. Like, if they’re having challenges emotionally with depression or stress or whatever it may be, it may be the fact that they don’t have income or the fact they don’t have a job or they don’t have housing- is contributing to their stress or their depression or anxiety, or whatever it may be that they may be having challenges with. I would say because of that, PRAPARE is definitely in line with that because it impacts the overall, I guess, challenges with that patient. That’s what I’ve been seeing.” (African American female MSW, LCAS-A Case Manager with 4.5 years of practice experience; *Facilitator*)	“Yeah…when you have people that actually listen—that was another thing about the social worker, she actually listened to me.” (26 year old African American male patient; *Facilitator*) “Yeah, I was comfortable because of their approach. You know, some people have a hard approach, you know, to you, to your situation—you know, why this happen, why that happen? But they didn’t go into that. They didn’t go into, “What did you do with every penny?” You know, because I told her, I said, “Look, I only get so much a month, I only get SSI, and I have a high gas, high electric.” So she was like, “Don’t worry about that. This is what you need.” You know?” (64 year old African American female patient; *Facilitator*)
Inner and Outer Context	“Honestly, I think that’s a double-edged sword because we’re not dealing with patients – and I hate to say it, I’m not trying to be biased – but we’re not dealing with <local health system> patients. All of our patients, pretty much, are coming with complex case management needs. And, it’s gotten worse with COVID. There are 40,000+ patients at <FQHC name>, who have these complex case management needs. And, there’s four case managers. So, how do you expect the limited number of people to be able to give these patients the quality that they deserve to address those needs?” (White/Caucasian female MSW, LCSW, LCAS-A Case Manager with 4 years of practice experience; *Barrier*)	“The Housing Authority is failing the tenants. They are. They really are. And I’m like, I just don’t get it. I don’t get it.” (64 year old African American female patient; *Barrier*) “I set an appointment, I cancelled an appointment, and I feel that they don’t want to treat me. I don’t know why. I cancelled the last appointment because I have fever. It’s very difficult to go to the dentist with fever. I don’t know why they are not flexible. And as I told you before, they are dealing with patients, they are not dealing with machines or robots.” (43 year old Hispanic male patient; *Barrier*) “I had to go to the social service department and provide proof. It was just such a long process and they didn’t need an appointment, but I was there for like hours and it was so draining. I’m like, if I had known this, I would have just made an appointment, maybe it would have been faster. I sat in the lobby for a long time, probably like a good hour waiting to see someone… Do you know what I’m saying? I didn’t want to spend half of my day sitting there waiting to get assistance with a bill.” (48 year old African American female patient; *Barrier*)
Societal Influence	“…I can administer the paperwork. I can give people the resources. But, if resources aren’t there, then to me that diminishes the effectiveness of the PRAPARE because now, “Great, I got all this information. Guess what? I really don’t have much I can help you with,” specifically housing-wise or for the individuals that need help paying utilities but the system is not set up to help them, whether they’re not eligible for it or they can’t get the help because the bill is in somebody else’s name or… So, it’s effective to get the information, but where the resources aren’t, then it makes it ineffective. Where the resources are, it makes it very effective.” (White/Caucasian female MSW, LCSW, LCAS-A Case Manager with 4 years of practice experience; *Barrier*) “Yeah, I see it a lot, and then I don’t know if they- Because I have a lot of patients where they come in and they say, “I don’t wanna ask for anything because I don’t want them to think I’m trying to use the government and I’m trying to fix my papers, and if I use this then it’s gonna stop me from doing this. I don’t wanna use this.” And so, they automatically think that asking for food or asking for anything is gonna mess up that aspect.” (Hispanic female MSW, LCSW-A Case Manager with 3 years of practice experience; Barrier)	“I’ve always done everything for myself. So, when you’re done everything for yourself it is so embarrassing to beg. Not beg, but it’s—… Because you’re used to doing those things for yourself. So, because you’re not used to it, so it’s hard to just get up and start asking people and things like that. So, it was a little difficult to ask about it.” (62 year old African American female patient; *Barrier*) [Did you find that anything else, just like in your background, your culture, that made it difficult to ask those questions or reach out for help?] “Yes. Because, like my culture—when we come, and you travel, you travel to be strong. You don’t travel to be weak, because we are stronger like 10 times more because that’s why we came here. We didn’t come to be a liability, you see? So, these are some of the things that you think about, too. You didn’t come to be a liability, and so we work like 10 times harder.” (62 year old African American female patient; *Barrier*) “The problem—let me be honest with you. We are in the process with the United States Citizenship and Immigration Service, and I told the person, I remember the—probably she was a social worker or something like that. I told the person that we can’t receive any, any help from the government right now because we are in the process with the United States Citizenship and Immigration Service. I talked with my lawyer. He told me that it’s not the time—it’s not good for us to receive any government help right now.” (43 year old Hispanic male patient; *Barrier*) “Sometimes, when you’re applying for a job, or when you are filling out a form, sometimes I feel that it’s like discriminatory. Discriminatory because if, for example, if I say ________, “He’s Hispanic, he’s American Indian,” probably you don’t get that job, or you don’t get—or probably, sometimes—I think sometimes that you don’t—you’re going to be treated fairly or something like that because it’s a barrier.” (43 year old Hispanic male patient; *Barrier*) “People judge. You know, “Why do you need help? What did you do with—aren’t you working?” Things like that. Because people tend to judge. If you’re in need and they figure it’s something you didn’t do right or it’s something you did wrong, whereas that may not necessarily be the situation.” (48 year old African American female patient; *Barrier*) “Well, social needs, financial needs, I don’t discuss with anybody because ain’t nobody going to listen to you. The people that I normally talk to, they don’t have no more than I have, so there’s no one else to talk to because don’t nobody else want to listen. That’s how I look at it. Like the mayor. We talk and talk and talk and talk, and he just does what he wants to do.” (62 year old African American female patient; *Barrier*)

### Social needs screening

Social needs screening included clinical activities related to patient identification, administration of the PRAPARE assessment, and EHR documentation.

#### Characteristics of the innovation and clinical encounter

According to patients, important facilitators included not feeling rushed, yet also having the PRAPARE administered in a short period of time (5–20 min) by a behavioral health case manager after a warm hand-off from their primary care provider as part of their regular medical visit. The convenient time frame and team-based approach were widely reported by patients as increasing the value and convenience of the medical visit. Clinician respondents, however, indicated that EHR documentation could present a time-cost burden when added to clinical responsibilities and suggested that auxiliary members of the care team could handle documentation tasks to extend clinicians’ bandwidth for seeing and assisting patients.

Patients perceived their relationships with clinicians who built rapport through respectful listening, empathic communication, motivational interviewing, and shared decision making as a facilitator to the screening process. One patient described the value of empathic communication by contrasting it with a negative experience at a local social service agency:

*“I was comfortable because of their [the clinician’s] approach.*. *.. [S]ome people have a hard approach. .. to you, to your situation. .. I said, “Look, I only get so much a month. I only get SSI, and I have a high gas, high electric.” She was like, “Don’t worry about that. This is what you need.”* -64-year-old African American female patient.

Although patients reported feeling comfortable and willing to talk about their social needs, positive interactions with clinicians were crucial to initiating a conversation to ask for assistance. One patient respondent said,*“I’ve never dreamed of asking anybody until she [the clinician social worker] approached me that day.”* Another patient appreciated that a member of the health care team had initiated the discussion given the stigma associated with seeking assistance for social and financial needs:

*“She [the social worker] stressed that ‘You just need to ask,’. .. that was what I really remember, so it took the shame off my face … it is so embarrassing to beg … you’re used to doing those things for yourself. .. it’s hard to just get up and start asking people [for assistance].”* -62-year-old African American female patient

Patients were asked to comment on screening protocols that included proactive outreach and universal screening. They reported that they would feel comfortable using alternative modalities including self-screening, patient portal messages, e-mail, or text messaging; however, they felt there were advantages to the in-person, interview approach to screening. As one patient commented, *“I like the one-on-one, being it’s more personable.”*

#### Patient & Provider Factors

Patient implementation factors included perceived acceptability, appropriateness, and community or engagement preferences. Patients viewed social needs screening as not only acceptable but an important component of high-quality primary care. They appreciated that members of their care team cared about their social and economic situations since, in their experience, these were not typically discussed during medical visits. One patient indicated that the screening made her feel that her provider cared for her *“as a whole,”* was interested in her *“personal life,”* and could provide better care because the care team understood what she was going through. Another patient felt their visit became more productive when their provider learned that economic pressures posed by expensive medication made adherence to treatment plans difficult and thus tailored referrals to resources and social services. Many patients indicated that such experiences were in stark contrast to previous medical visit experiences in which, *“you just went for treatment. They treated you and that was it;”* patients had not realized that a range of additional support could be provided. Patients reported that open discussions of social and economic needs had improved their relationship with their health care team. No patients indicated concerns about privacy or the health care team’s access to information in their EHR.

The methods and beliefs of clinicians when screening for social needs as a component of care planning were important implementation facilitators. Clinicians described that use of the PRAPARE was aligned with their professional goals to provide a comprehensive and systemic evaluation of patients’ co-occurring social needs. One clinician noted that the PRAPARE showed, *“how the different parts of a person’s life impact them because. .. one area influences the other.”*

Clinicians also reported that specific, evidence-based patient engagement techniques, such as empathic communication and motivational interviewing, facilitated implementation and delivery of the screening assessment. One clinician described how reflective listening and reframing improved understanding of patients’ needs and empowered the patients during the PRAPARE interaction:

*“[T]he reflection allows. .. the patient to feel that they are being heard, and you’re also allowing yourself to check in to make sure you’re hearing the patient correctly.. .. [T]he reframing is. .. strength-based, positive-based, to empower the patient.”*- Hispanic female Case Manager

#### Inner & outer context

Clinician case managers felt that the alignment of social needs screening with the role of health care organizations and the ethos of patient-centered care were important implementation facilitators. Clinicians noted the role of leadership engagement as a critical dimension of implementation. Incorporating front-line clinician and staff perspectives on decisions related to identifying priority patient populations, workflows, compatibility with current workload, design of the screening assessment, and quality improvement activities contributed to the implementation effectiveness of the screening protocol.

#### Societal influence

Clinicians expressed that while the PRAPARE provided a *“formal” or “structured and standardized way”* of assessing patients’ needs, follow-up was needed to ensure that needs were mitigated or resolved. One clinician noted that, *“there’s no definite end for it.. .. how is it helping the patient, besides identifying that there is a problem?”* Another alluded to the need for ongoing support for patients with complex social needs: “*I see some benefits. .. but it’s definitely not an easy project in general.”* Case managers expressed concern about unprofessionalism and emotional trauma attached to asking patients to discuss domains on the PRAPARE assessment for which they could provide no assistance or for which resources were lacking, and found it particularly difficult to justify administering some PRAPARE screening items (e.g., housing) without evidence of substantial, longer-term benefits.

Clinicians reported that awareness of implicit and explicit biases and problem solving were helpful clinical techniques. One clinician noted*:*

*“Knowing your own biases right away [is important] because you can get triggered with some of the response[s] patients give, and that will affect your rapport with them or how you respond.”* - Hispanic female Case Manager

Both clinicians and patients also reported that patients sometimes experienced stigma associated with societal norms surrounding needing or asking for help and empathetic rapport can help patients be more receptive to being screened and accepting assistance.

### Social needs response and case management

Social needs response included clinical activities related to the referral of patients to internal resources, social service, agencies, or community-based organizations, including additional case management or follow-up for patients who accepted a referral.

#### Characteristics of the innovation and clinical encounter

Both patients and clinicians expressed that detailed and accurate information about community resources facilitated resolutions of referrals. One patient respondent noted, “It was put together perfect for me, because I didn’t have to *scramble around. I didn’t have to call 10 or 15 different places.”* Conversely, inaccurate or dated information may be a potential barrier to the referral and response component of the PRAPARE.

Rapport between patients and clinicians facilitated the often lengthy and bureaucratic process of contacting referred resources to initiate services. Patients appreciated that clinicians were proactive during the PRAPARE encounter:

*“It kind of shocked me because some social workers have said, “Well, maybe in a few days, I’ll call,” or. .. “Hopefully I’ll get around to it,” but [my clinician said,] “Let’s get on this right away.. .. I’m going to try to call it.” And I was really shocked. It really touched my heart.” -*64-year-old African American female patient

Clinicians expressed the importance of developing trust to enable clinician-patient problem solving. One clinician commented:

*“Sometimes the patient is like, “I don’t know. .. We haven’t talked in so long.” But then, we’ll call the brother together, and [the brother is] like, “Oh, I didn’t know this was what was going on with you. Why didn’t you tell me?”* – African American female Case Manager

#### Patient & Provider Factors

Clinicians noted that unmet social needs rarely appeared in a vacuum; they often interconnected with medical needs, creating complex challenges for patients’ individual situation and their clinician’s response. For example, one patient identified financial barriers affecting their health care access, a situation exacerbated by lack of transportation. Another patient described an unmet medical need that led to unemployment: *“My managers would see me. .. fall, and they would say, ‘We’re going to have to lay you off till you get. .. your knees taken care of.’”* The cumulative impact of intersecting unmet social needs often complicated the referral of patients to resources. For example, one clinician case manager noted that patients without reliable transportation had difficulty visiting the local food bank to access services to address food insecurity.

We found that knowledge of community context was a facilitator to implementation. Relevant local information allowed case managers to respond to the nuances and complexities of their patients’ identified social needs. One patient described the value of their newly-acquired awareness of resources after their PRAPARE encounter: *“There’s so many. .. [resources] around this area that you don’t know. .. exist until you’re in need or someone cares about the need.”*

#### Inner & outer context

Clinicians reported that staffing constraints were a significant barrier to expanding the reach of social needs S/R. In particular, significant variation in patient response activities was not always accounted for in the organization’s consideration of scheduling and panel size. One case manager noted,

*“There are other things going on that aren’t getting counted.. .. we might do four PRAPAREs and it looks like two hours on our schedule, but really all of the complex case management needs and coordinating. .. may have taken us six hours.” -*White female Case Manager

Another case manager added,

*“We’re being divided everywhere, and that’s not. .. being taken into consideration.. .. We’re not superheroes. We can try. .. but at the end of the day. .. there’s no recognition for everything that we are doing to be those best social workers for our patients and get them what they need.” -*White female Case Manager

To address this, clinicians suggested using a *“lighter-touch”* model for patients with lower social and medical complexity and a more intensive approach for patients with urgent social needs or in crisis.

Clinician case managers reported that their case management activities were not always coordinated with other health systems. Duplicated efforts and fragmentation between the FQHC and the larger integrated health system could lead to patient dissatisfaction and confusion. As a solution, one patient suggested that hospitals and health systems use a “unified system” or “data center” as a data-sharing infrastructure for better coordination around social needs.

#### Societal influence

Both patients and clinicians described implementation considerations shaped by structural economic and social forces and policy-level determinants. For example, patients screening positive for housing insecurity were confronted with a limited supply of affordable housing and lack of funding for programs designed to provide housing assistance. One respondent described the difficultly of accessing housing through the local housing authority: *“The [person]. .. from the Housing Authority when I was calling was like, ‘We’re no longer taking Section 8.’”* Another patient expressed, *“the Housing Authority is failing the tenants,”* adding that their trust in the institution had been undermined by exploitative landlords who did not make basic repairs. Case managers corroborated the reports of difficulties with housing resources and emphasized that, in general, the effectiveness of the PRAPARE depended on the availability of appropriate resources. They described the PRAPARE screening as akin to opening *“Pandora’s box”* in that it often disclosed complex patient needs that case managers were unable to address. As one case manager remarked: *“If you don’t have the resources to help,. .. what do you tell the patient?”* Clinicians also described how the COVID-19 pandemic had exacerbated social needs and complicated efforts to respond to them:

*“People. .. are accustomed to just going in and. .. talking face-to-face. .. There’s a lot more stress because they can’t get a hold of people, of resources – they’re waiting longer.” -*White female Case Manager

Administrative burden presented a significant implementation barrier, hindering efforts to refer patients to needed social services or establish effective referral pathways. One patient described challenges related to means testing and layers of eligibility criteria:

*“It’s crazy. In the last year, I’ve needed help.. .. I probably would have gotten approved, but. .. all of the hoops and hoops you have to jump through and. .. over to get it. I just wasn’t in for it, so I didn’t bother.*” -48-year-old African American female patient

Another patient described administrative and eligibility requirements that created tense and adversarial relationships with social service agencies:

*“They [Social Service Department] be like, “Well, okay,. .. you got $60 left. What did you do with that?”. .. I have to buy food.. .. if I try to shop on the diabetic aisles, diabetic food is higher. .. it’s more expensive. So, they don’t look at it like that. They won’t accept the fact that you may have to pay out your pocket.”* -64-year-old African American female patient.

Clinician and patient descriptions of social and economic policies and procedures based on experiences with administrative burden and under-resourced social services were representative of structural inequities.

Patients reported experiencing discrimination and racism when accessing resources and during encounters with the larger health care system, social service and transit agencies, and/or community-based organizations. These experiences were barriers that discouraged participation and undermined trust in health care and social service institutions.

## Discussion

Patient and clinician perspectives are critical to inform the implementation of social needs S/R that complements the ethos of the patient-centered medical home to improve outcomes and promote health equity. Guided by the HEIF, we identified patient and clinician perceived barriers and facilitators to implementing the PRAPARE with a patient population that has historically experienced economic exclusion, discrimination, and marginalization. Four key points emerged with practical implications.

First, patients and clinicians found the S/R approach acceptable and supported its implementation as an element of comprehensive, high-quality care. Patients and clinicians viewed social needs screening as consistent with the ethos of patient-centered care in the medical home, which emphasized ‘whole-person’ care through responsiveness to patients’ values, preferences, and needs. These findings are largely consistent with previous research [30, 32, 51, 52]. For example, De Marchis et al. and Byhoff et al’s two-part evaluation of social needs screening acceptability among patients found that patients were comfortable with the screening process and thought it was an important element of empathetic, patient centered care [51, 52]. Similarly, we found that patients did not report privacy concerns as a barrier and believed there were benefits to broader screening, including spillover benefits for care planning and treatment of medical needs. Many patients were amenable to being screened through alternative modalities (electronic message, text message, or self-screening during intake), but some expressed that talking in person was more effective to elicit sensitive information and overcome unwillingness to discuss nonmedical needs.

Second, clinicians reported that staffing constraints within the health center presented significant barriers. To extend the bandwidth of clinicians and promote greater reach of social needs S/R, our findings suggest that social needs S/R implementation efforts may be bolstered by more sustainable staffing models or alternative delivery models that leverage existing technologies. For example, to complement reforms to the state Medicaid program, North Carolina has invested in the first statewide network to integrate health care and human services organizations by using a shared technology platform to send and receive electronic referrals, communicate in real-time using text and chat capabilities, and track client outcomes. Investments in technologies may improve implementation effectiveness of social needs S/R protocols [53].

Third, social and medical needs are clustered and interrelated, suggesting that response protocols must be tailored to address commonly co-occurring clusters and reflect the reality of the lack of funding for or availability of social services or community-based resources. For example, unemployment and the inability to access health services due to financial burden commonly co-occur and require a comprehensive response [54–56], such as complementary referrals and case management across interrelated social needs. Implementation efforts should coordinate social needs response protocols with ongoing care planning to ensure that they are complementary to treatment goals and newly identified social needs, with special consideration for patient populations with common chronic illnesses like diabetes [57]. Patients and clinicians noted that shared decision-making, empathic communication, and accurate information about community resources or social services could facilitate personalized care. Patients described the effectiveness of a screening and shared decision-making process wherein they could choose from community-based resources or social services based on location, hours, eligibility criteria, and preference or priority.

Fourth, our findings also suggest that implementation strategies within the vertical structure of a health system alone cannot overcome the broader administrative and economic policies that hinder access; this includes, but is not limited to, the administrative bureaucracy of social service delivery. Within the HEIF *Societal Influence* domain, we found that upstream determinants of health are especially challenging to address through individual-level intervention and require intervention at the system or policy level (e.g., simplify application processes, address administrative burden, expand funding, reduce means testing). Patients reported that mistrust of medical and social service institutions was a barrier to responding to clustered and interrelated social and medical needs, suggesting that implementation efforts must recognize institutional mistrust and design context-specific strategies. Vulnerable communities can be better served by using strategies such as multisector coalition building around health and social care integration to coordinate and advocate for policy-level changes [11, 53, 58, 59]. For example, the administrative burden of applications to social service and entitlement programs can be reduced, harmonized, or streamlined to promote uptake. This type of systems-approach is consistent with Health In All Policies, an overarching public health framework which posits that health promotion considerations should inform all policy decisions that influence social, political, and ecological health determinants [60].

When combined with previous literature, our results suggest that translation efforts must include (i) an overarching strategy that identifies community-specific opportunities for and barriers to health equity [61, 62], (ii) development of an explicit linkage between social needs S/R protocols and a population health management strategy for underserved patient populations, and (iii) evaluation of implementation strategies according to performance measures associated with health equity. These findings should be interpreted within the context of several limitations. The sample size was appropriate for the research question of interest but may limit generalizability to other contexts, workflows, and populations (e.g., unique barriers and facilitators for immigrant and undocumented populations or experiences of other clinical stakeholder perspectives including primary care providers). Additionally, although we took steps to encourage honest answers, there may have been social desirability bias given the sensitive subject matter. Finally, the nature of the academic-community partnership and the ongoing evaluation may have influenced the implementation process.

Our study has several strengths that contribute to the growing body of literature informing strategies for increasing the uptake of the PRAPARE and S/R approaches like it. To our knowledge, it is the first to elicit in-depth patient and clinician perspectives on multi-level barriers and facilitators to implementing patient-centered protocols for identifying and responding to social needs. This is an area of interest to both interventionists and implementation scientists. Our research contributes to the development of generalizable implementation strategies to promote the uptake of social needs S/R as part of routine care delivery in diverse settings. Implementation strategies must be designed to be adaptable and may include clinician training and workforce development to utilize shared decision making, EHR-integrated decision support tools [28, 43], or the formation and coordination of multi-sector partnerships to identify best practices for social needs S/R models that strengthen the medical home, inform population health management and advocacy efforts, and improve outcomes associated with high quality care and health equity. As S/R interventions become increasingly common, it is important that implementation scientists and health services researchers advance several priority research areas to ensure effective translation into real-world clinical settings. We suggest that further research is needed to (i) identify potential areas of symmetry between clinicians’ perspectives on barriers and facilitators and those of their patients, (ii) compare modalities for social needs screening to increase reach in a patient-centric manner, (iii) understand distinct implementation considerations relevant to special populations such as immigrants or refugees, and (iv) identify the most effective response or social care interventions to realize the most significant improvements in health outcomes, equity, and cost.

## Conclusion

Screening and responding to social needs are essential mechanisms for delivering equitable, patient-centered, and value-based care. Careful consideration of implementation factors is required to integrate changes associated with social needs S/R into the demanding workflow of the primary care clinical environment. We identified individual encounter, organizational, community, and societal level factors that have practical implications for implementing the PRAPARE and or similar social needs S/R protocols into primary care. The HEIF was suitable for identifying barriers and facilitators to equitable implementation in this dynamic clinical environment. Future research should focus on the selection of implementation strategies to promote social needs S/R and associated multisector care coordination responsive to the implementation factors identified.

## Supplementary Information

**Additional file 1.** Patient Semi-Structured Interview Guide. Questions were designed to evaluate patient-reported barriers and facilitators across the HEIF domains.

**Additional file 2.** Clinician Focus Group Guide. Questions were designed to identify areas of consensus and disagreement across the HEIF domains.

## Data Availability

The de-identified data analyzed for the current study are available from the corresponding author upon request.

## Abbreviations

CBOs: Community based organizations
EHR: Electronic health record
FQHC: Federally qualified community health center
HEIF: Health Equity Implementation Framework
i-PARIHS: Integrated-Promoting Action on Research Implementation in Health Services
PRAPARE: Protocol for Responding to and Assessing Patients’ Assets, Risks, and Experiences
SDOH: Social determinants of health
S/R: Screening and referral
SRQR: Standards for reporting qualitative research
WIC: Women, infants, and children
