# Supplementary material for: Implementation of social needs screening in primary care: a qualitative study using the health equity implementation framework
Source: BMC Health Serv Res. 2021 Sep 17;21:975. doi: 10.1186/s12913-021-06991-3 (PMC8445654; doi:10.1186/s12913-021-06991-3)
Supplement: Supplementary file 1 — Additional file 1. Patient Semi-Structured Interview Guide. Questions were designed to evaluate patient-reported barriers and facilitators across the HEIF domains. [file 12913_2021_6991_MOESM1_ESM.docx]

**Patient Experience related to the Protocol for Responding to and Assessing Patients’ Assets, Risks, and Experiences (PRAPARE) at [FQHC]**

**Patient Semi-Structured Interview Guide**

**Purpose:** To understand the patient experience of PRAPARE

**Respondents:** A [FQHC] patient that has participated in PRAPARE

**Anticipated Time:** 60 minutes per interview

Subject ID: ______

Age:

Gender:

Race:

Education level:

Living situation:

PRAPARE SDOH Risk Factors Identified:

Community Resource Referrals:

Start Time: _________________________AM / PM

End Time: _________________________AM / PM

Interviewer: _____________________________________________________

Thank you very much for your time and participation today. Let’s talk about your experience at [FQHC]. During your visit with (behavioral health case manager name) back in (month of PRAPARE administration) you were asked questions about non-medical needs like food, housing, stress, and transportation. *[Hand patient PRAPARE Sample and leave out during the interview to reference as needed].* Here is a sample of the questions that you were asked, such as…[*read out a few of the questions*]. Then, once you gave your answers, you were provided resources in the community *[list community resource referrals that were made].* I want to learn more about your experience answering these questions and receiving referrals to these community resources so that we can improve the way health care can support patients and provide better quality care.

If it’s okay with you, I’d like to digitally record this interview. The recording will be kept on this password protected recorder until I can download the recording into a secure, limited access folder that only myself and the study team has access to. After the recording is downloaded, it will be immediately deleted from the recorder. We will then send it to a [IRB] approved transcription service that will transcribe the interview for us so that we can learn more about your experience. Do you have any questions about that process? *[Wait for response, if no questions, continue on with the interview].*

If you’d like me to stop recording at any time, please let me know and I’ll turn off the recorder. Also, please remember that you can always decline to answer any of my questions. Your responses will be kept completely confidential and will not affect your care at [FQHC] or from any community organization. Do you have any questions before I turn on the recorder? *[Wait for response, if no further questions, begin the recorder and the interview questions].*

**I. Characteristics of PRAPARE Assessment and Referral (*HEIF Framework Element: Characteristics of the Innovation)***

1. PRAPARE stands for the Protocol for Responding to Patients Assets Risks and Experiences and includes questions on social and economic aspects of your life. For example, whether you have stable housing, enough food for you and your family, and about your employment situation. Why do you think you were asked these questions?
   - *Probe:* Are there other aspects of your life that you think it’s important for your health care team to know about that are not covered in the questions you were asked?
   - *If yes:* Please describe to me what some of those aspects are.
2. Do you think it is important that [FQHC] has this information? Why or why not?
   - *If yes:* In what ways do you think these topics are important to your health?
   - *If no:* Why do you think these topics are not important to your health? What topics would be important for us know about your health?
3. What do you remember about the conversation you had with [NAME] about your social and financial needs?
   - *Optional Probe:* What was the most memorable part of your experience in answering these questions with [NAME]?
4. Approximately how long did it take to answer these questions with [NAME]?
   - *Optional probe:* Could you tell me more about the amount of time it took?
5. What do you remember about the community resources you were provided?
6. Was it hard to access the community resource you were referred to?
   - *Optional follow up probing question:* Could you tell me more about why it was hard/easy?
   - *If no*, how did you decide on which resources that you would look into?
   - *If yes,* what could have made it easier for you to look into the resources you were provided?
7. Did you feel that the community resources you were referred to were helpful?
   - *Optional follow up probing question:* Could you tell me more about why these resources were helpful/unhelpful?

**II. Patient Experience (*HEIF Framework Elements: Clinical Encounter, Patient Factors, Provider Factors)***

*Thank you for sharing all of that information with me. Now I’d like to learn more about what made the PRAPARE process easier or harder. Are you ready?*

1. Were you comfortable sharing answers with the staff member at [FQHC]? Why or why not? What could have made the experience even better?
2. Were any of the questions difficult to understand?
   - *Optional follow up probing question:* Could you tell me more about why it was hard/easy?
3. Did you have any concerns about your privacy?
   - *Optional Probe:* How do feel about your responses to [NAME] questions being shared with other members of your health care team?
   - *Optional probing question if response is in the affirmative:* Could you tell me more about your privacy concerns?
4. We are considering administering the questions [NAME] asked you in different ways.
   - What are your thoughts on answering these questions using a paper form in clinic while you wait?
   - What are your thoughts on answering these questions online using a computer, phone, or tablet?
   - What are your thoughts on answering these questions over the phone?
   - How could we improve your experience of answering the questions in person with a [FQHC] staff member?
5. We are considering different ways to follow up with patients after they’ve answered the questions and received community referrals. The goal of a follow-up would be to make sure patients were able to access the community resources, and if not, provide a reminder, information (i.e., hours of operation, contact information, forms), or help troubleshoot challenges to accessing the resource.
   - What are your thoughts on receiving a follow-up call from a volunteer or member of the health care team at [FQHC]?
   - What are your thoughts on receiving a follow-up text message?
   - What are your thoughts on receiving a follow-up email or message through your patient portal [ex: MyChart]?
6. If you had any, did you feel that your questions were answered by [NAME]?
7. Did you feel like you were treated differently or unfairly while answering these questions with [NAME]?
   - *Optional follow up probing question:* If so, could you tell me more about this experience?
8. Did you feel that you needed more time with [NAME] while answering the questions?
9. Did you feel that you were respected by [FQHC] staff or clinicians during your visit where you answered these questions [can reference the physical PRAPARE form]?
   - *If no*: Please describe why you felt this way.
10. Did you feel that [NAME] gave you choices or accepted your input on the type of community resource to use?
    - *Probe*: How was your input utilized in choosing community resources?

**III. Context and Health System Factors (*HEIF Framework Elements: Inner Context, Outer Context)***

*Thank you for sharing all of that information with me. Now I’d like to learn more about your experiences with health care and in the community in general. Are you ready?*

1. Had you previously ever had a bad health care experience?
   - *Optional follow up probing question:* If so, could you tell me more about that experience? Do you think it impacted your experience with PRAPARE?
2. Had you ever had a bad experience at a community resource before?
   - - *Optional follow up probing question: If so, could you tell me more about that experience?* Do you think it affected your experience with PRAPARE?
3. Did you feel that there is an expectation from society that you not accept help?
   - - *Optional follow up phrasing:* Do you feel like there’s an expectation you shouldn’t accept help? Did you feel uncomfortable accepting help?
     - *Optional follow up probing question:* If so, could you describe how this may have affected your experience with answering PRAPARE questions about your social and financial situation?
   - *Optional follow up probing question:* If so, how did this effect your willingness to accept the referral to this community resource?
4. Did you feel that there is an expectation from society that you not discuss social or financial aspects of your life?
   - - *Optional follow up phrasing:* Do you find it hard to discuss social or financial aspects of your life in a doctor’s visit?
     - *Optional follow up probing question:* If so, could you describe how this may have affect your experience with answering PRAPARE questions about your social and financial situation?
     - *If affirmative, follow up question:* Would you mind sharing what made it difficult?
5. Do you feel your identity (ex: race, gender, religion, etc.) has led you to be treated differently in a health care or community settings? For example, did you feel that your race, gender, or identity impacted how you were treated?
   - *If yes,* Tell me more about this. How were you treated differently?
   - *If yes,* What could we do to further improve how you are treated here?

Thank you for your time!

*[Stop audio recorder and as soon as possible load recorder to secure drive. Once successfully saved to drive, delete recording from recording device.]*
