# Supplementary material for: Implementation of social needs screening in primary care: a qualitative study using the health equity implementation framework
Source: BMC Health Serv Res. 2021 Sep 17;21:975. doi: 10.1186/s12913-021-06991-3 (PMC8445654; doi:10.1186/s12913-021-06991-3)
Supplement: Supplementary file 2 — Additional file 2. Clinician Focus Group Guide. Questions were designed to identify areas of consensus and disagreement across the HEIF domains. [file 12913_2021_6991_MOESM2_ESM.docx]

**Case Manager Experiences related to the Protocol for Responding to and Assessing Patients’ Assets, Risks, and Experiences (PRAPARE) at [FQHC]**

**Case Manager Semi-Structured Focus Group Guide**

**Purpose:** To understand the case manager experience with PRAPARE

**Respondents:** Case managers at [FQHC] who administer PRAPARE

**Anticipated Time:** 60 minutes

Subject ID: ______

Age:

Gender:

Race:

Education (degrees, certificates, and other licenses):

Languages:

Years of Practice:

Years at [FQHC]:

Sites at [FQHC]:

Training/Licensure:

Start Time: _________________________AM / PM

End Time: _________________________AM / PM

Interviewer: _____________________________________________________

**0. Introduction**

Welcome all! Thank you so much for your time and participation today. First and foremost, we are so grateful for your effort administering the Protocol for Responding to and Assessing Patients’ Assets, Risks, and Experiences (PRAPARE) here at [FQHC]. Last spring, we interviewed ten patients to hear their perspectives on receiving PRAPARE, and many of them were similarly appreciative of your attention and care.

We want to ask all of you about your perspectives after several years of experience with PRAPARE at [FQHC]. This includes your perspective on the process related to initial screenings, making referrals, and following up with patients. Your feedback will help us have a fuller picture when we are evaluating the program and thinking about next steps for the future.

In today’s conversation, my role as moderator will be to guide the discussion. ([name] will be assisting by taking notes and managing any technical difficulties.) Keep in mind that we are interested in both positive and negative comments about PRAPARE, and please feel free to share your point of view even if it differs from what others have said. The same goes for feedback about its implementation at [FQHC]—both positive and especially negative comments will help us improve moving forward. And, please feel free to talk and respond to one another, rather than just me.

If it’s okay with you, I’d like to digitally record this conversation. The recording will be kept on this password protected recorder until I can download the recording into a secure, limited access folder that only myself and the study team has access to. After the recording is downloaded, it will be immediately deleted from the recorder. We will then send it to a [IRB] approved transcription service that will transcribe the audio for us so that we can learn more about your experience. Do you have any questions about that process? [Wait for response, if no questions, continue on with the focus group].

If you’d like me to stop recording at any time, please let me know and I’ll turn off the recorder. Also, please remember that you can always decline to answer any of my questions. Your responses will be kept completely confidential and will not affect your roles at [FQHC]. Do you have any questions before I turn on the recorder? [Wait for response, if no further questions, begin the recorder and the questions].

**I. PRAPARE Intervention & Processes (HEIF: Characteristics of the Innovation, Clinical Encounter)**

[*Screen-share PRAPARE Sample and leave it up during the focus group to reference as needed.*]

1. What are your goals in administering PRAPARE with patients?
   - *Optional follow-up:* How aligned is PRAPARE with your goals and purposes as a case manager?
2. How effective do you believe administering PRAPARE has been for meeting the needs of patients?
   - *Optional follow-up:* What are measures of efficacy or success that you look for?
   - *Optional follow-up:* What difficulties do your patients encounter in the screening and referral process?
3. How convenient has the PRAPARE tool been for screening and referring patients?
   - *Optional follow-up:* What could make your experience administering PRAPARE easier?
4. How convenient has it been to incorporate PRAPARE into your clinical workflow?
   - What are your thoughts on administering PRAPARE through a self-screener, such as the model proposed for universal screening? What about through texts or MyChart?
5. How helpful has the student-volunteer Help Desk been in supporting your goals?

**II. Patient-Case Manager Interactions (HEIF: Patient Factors, Provider Factors)**

1. In general, how receptive are patients to the process of administering PRAPARE?
   - *Probe:* What are common barriers that patients face?
2. What are the two or three most helpful personal traits, skills, or communication strategies for administering PRAPARE with patients? (e.g., inquiry, empathic communication, or shared decision making)
   - *Probe:* Can you give an example of this in action?
   - *Probe:* Imagine you were advising a new case manager who will be administering PRAPARE. What would you warn them *not* to do when screening and referring patients?
   - *Optional probe:* What strategies or communication techniques do you employ to create a trusting environment for patients to answer your questions and reduce the stigma of seeking help?
   - *Optional probe:* What strategies or communication techniques do you employ to motivate patients to overcome the barriers they face and listen to your guidance?

**III. Context and Health Systems Factors (HEIR: Context, Societal Factors)**

1. How do you manage needs or referrals that are more difficult to resolve, for structural reasons? For example, historically it has been difficult to help patients find housing, and often housing organizations are not as helpful or immediate as, let’s say, food pantries.
   - *Follow-up:* What could be done to improve PRAPARE effectiveness for immigrants, refugees, and patients who do not speak English?
2. *Optional, time-permitting:* What kinds of external factors, whether in Durham or at the state or federal level, influence the implementation of PRAPARE at [FQHC]?
3. Think about the work culture, leadership and organizational structure, and relationships with external organizations. What kind of support would you like or need to implement PRAPARE better at [FQHC]?
   - *Optional follow-up:* What characteristics of [FQHC] do you think have been helpful for implementing PRAPARE?

**IV. Wrap-Up and Conclusion**

1. Thinking back on everything we discussed today, what are three things that we should take away from this discussion about your experience with implementing PRAPARE?
2. [summarize entire discussion and purpose of focus group briefly] Is this an adequate summary? Have we missed anything?

**V. [PLATFORM] Sub-Section (*Optional, time-permitting)***

1. How helpful has [PLATFORM] been for patients?
2. How helpful has [PLATFORM] been for you?
3. What do you believe needs to happen before [PLATFORM] can be adopted at [FQHC] and in [city]?
